# Clinical application of targeted next-generation sequencing for colorectal cancer patients: a multicentric Belgian experience

**DOI:** 10.18632/oncotarget.25099

**Published:** 2018-04-17

**Authors:** Nicky D’Haene, Quitterie Fontanges, Nancy De Nève, Oriane Blanchard, Barbara Melendez, Monique Delos, Marie-Françoise Dehou, Calliope Maris, Nathalie Nagy, Emmanuel Rousseau, Josse Vandenhove, André Gilles, Carine De Prez, Laurine Verset, Marie-Paule Van Craynest, Pieter Demetter, Jean-Luc Van Laethem, Isabelle Salmon, Marie Le Mercier

**Affiliations:** ^1^ Department of Pathology, Erasme Hospital, Université Libre de Bruxelles, Brussels, Belgium; ^2^ Department of Pathology, CHU UCL Namur, Yvoir, Belgium; ^3^ CMP Pathology Laboratory, Brussels, Belgium; ^4^ Department of Pathology, Braine l´Alleud Waterloo Hospital, Braine l´Alleud, Belgium; ^5^ Department of Pathology, Charleroi University Hospital, Charleroi, Belgium; ^6^ Department of Pathology, Mouscron Hospital, Mouscron, Belgium; ^7^ Department of Pathology, Sint Maria Hospital, Halle, Belgium; ^8^ Department of Pathology, EPICURA Hospital, Frameries, Belgium; ^9^ Department of Pathology, Brugmann University Hospital, Brussels, Belgium; ^10^ CurePath, Jumet, Belgium; ^11^ New LabPatho, Braine l´Alleud, Belgium; ^12^ Department of Oncology, Erasme Hospital, Université Libre de Bruxelles, Brussels, Belgium

**Keywords:** colorectal cancer, next-generation sequencing

## Abstract

International guidelines made RAS (KRAS and NRAS) status a prerequisite for the use of anti-EGFR agents for metastatic colorectal cancer (CRC) patients. Daily, new data emerges on the theranostic and prognostic role of molecular biomarkers; this is a strong incentive for a validated, sensitive, and broadly available molecular screening test. Next-generation sequencing (NGS) has begun to supplant other technologies for genomic profiling. We report here our 2 years of clinical practice using NGS results to guide therapeutic decisions.

The Ion Torrent AmpliSeq colon/lung cancer panel, which allows mutation detection in 22 cancer-related genes, was prospectively used in clinical practice (BELAC ISO 15189 accredited method). The DNA of 741 formalin-fixed paraffin-embedded CRC tissues, including primary tumors and metastasis, was obtained from 14 different Belgian institutions and subjected to targeted NGS.

Of the tumors tested, 98% (727) were successfully sequenced and 89% (650) harbored at least one mutation. KRAS, BRAF and NRAS mutations were found in 335 (46%), 78 (11%) and 32 (4%) samples, respectively. These mutation frequencies were consistent with those reported in public databases. Moreover, mutations and amplifications in potentially actionable genes were identified in 464 samples (64%), including mutations in PIK3CA (14%), ERBB2 (0.4%), AKT1 (0.6%), and MAP2K1 (0.1%), as well as amplifications of ERBB2 (0.3%) and EGFR (0.3%). The median turnaround time between reception of the sample in the laboratory and report release was 8 calendar days.

Overall, the AmpliSeq colon/lung cancer panel was successfully applied in daily practice and provided reliable clinically relevant information for CRC patients.

## INTRODUCTION

Colorectal Cancer (CRC) is the second most frequent cancer in Europe, irrespective of gender, and still yields a high mortality rate, accounting for 12% of cancer deaths [1]. Despite a broad screening program, 25% of the patients are metastatic at initial diagnosis. Moreover, one out of two patients will develop metastasis [1].

Current therapeutical guidelines for stage IV patients include a combination of cytotoxic and biological targeted agents [2]. The approved agents are monoclonal antibodies targeting epidermal growth factor receptor (EGFR): cetuximab and panitumumab. Until recently, indications for standard-of-care molecular testing in colorectal carcinomas included testing for KRAS mutational status as a predictor of response to anti–EGFR agents [3]. Now, American and European guidelines clearly emphasize expanded RAS (KRAS and NRAS) status as a mandatory precondition for use of anti-EGFR therapy [2, 4]. Indeed, not only is the benefit of anti-EGFR therapy confined to RAS wild type (wt) tumors, but treatment with anti-EGFR antibodies may even harm patients with a RAS mutation. BRAF mutation is a strong negative prognostic biomarker and evidence is accumulating that patients with a BRAF mutant tumor do not benefit from anti-EGFR therapy [2, 5].

Promising targeted therapy and personalized medicine are making molecular profiling of tumors a priority. International efforts to catalogue mutations for multiple forms of cancer, coupled with the successes of targeted agents in patients with molecularly defined tumors, have generated enthusiasm for incorporating genomic profiling into clinical cancer practice. Daily, new data emerges on the theranostic and prognostic role of molecular biomarkers. This is a strong incentive for a validated, sensitive and broadly available molecular screening test in order to implement and improve multi-modal therapy strategy and clinical trials.

We have recently validated and accredited (BELAC ISO 15189) the use of the Ion Ampliseq^™^ Colon and Lung cancer panel on the Ion Torrent Personal Genome Machine (PGM – Life Technologies) for screening lung and colorectal cancers. This NGS panel is a multiplex PCR-based library preparation method by which 90 amplicons that encompasses 1825 mutational hotspots of 22 genes related to colon and lung cancer are selectively amplified and sequenced.

In the present study, we evaluated the use of this panel on 741 samples from different institutions that have been tested in the context of daily practice since the accreditation of the technique.

## RESULTS

### Clinical series

**Figure 1:**
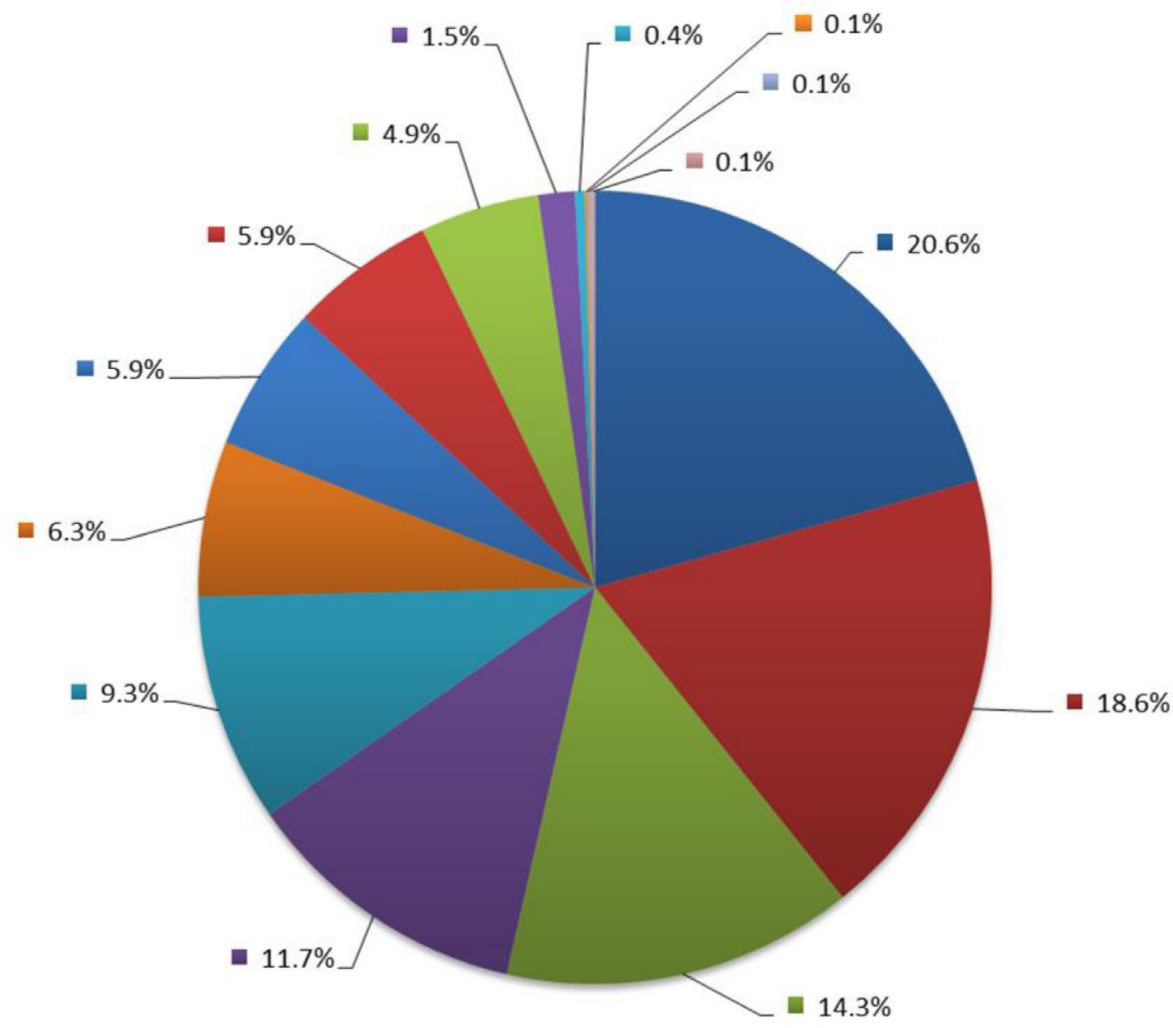
Pie chart of the distribution of institution contributions

A total of 741 FFPE samples from patients with colorectal cancer were received from 14 different institutions for NGS testing from November 2013 to December 2015, each contributing from 1 to 153 samples (Figure 1). For two cases (0.3%), sequencing could not be performed because of insufficient tumor tissue left. The type of the sample was recorded for 708 patients; the series included 390 surgical resections (55%), 311 biopsies (44%) and 7 cell blocks (1%). The site of the sample was recorded for 696 patients; the primary tumor was tested for 584 patients (84%) and metastasis for 112 patients (16%) (Table 1). According to international guidelines, either primary or metastatic specimens may be used depending on the particular case requirements [6, 7].

**Table 1:** Summary of the clinical series and sequencing performances

	Primary	Metastasis	Unknown	Total	
	*N*	*N*	*N*	*N*	(%)
**Primary Tumor/Metastasis**	584	112	45	741	
**Sample Types (*n* = 708)**					
Cell block	0	7	0	7	(1.0)
Biopsy	256	43	12	311	(43.9)
Surgical resection	316	58	16	390	(55.1)
**% of Tumor Cells (*n* = 735)**					
<10%	45	8	2	55	(7.5)
10–50%	463	62	34	559	(76.1)
>50%	71	41	9	121	(16.5)
**Sequencing Performance (*n* = 741)**					
Non-informative	8	6	0	14	(1.9)
Informative	576	106	45	727	(98.1)

### Sequencing performances

Sequencing performance was assessed from the number and distribution of sequencing reads across the targeted regions. Among the 741 sequenced cases, 727 (98%) were informative (Table 1). Average base coverage of all samples was 1,493×. Six cases with a coverage between 300 and 500× were also reported with a comment about the suboptimal coverage and the few exceptional cases (19) with average base coverage <500x and with relevant mutations that passed the quality criteria (Material and Methods) were identified. These mutations were reported because of their high clinical impact.

### Turnaround time

**Figure 2:**
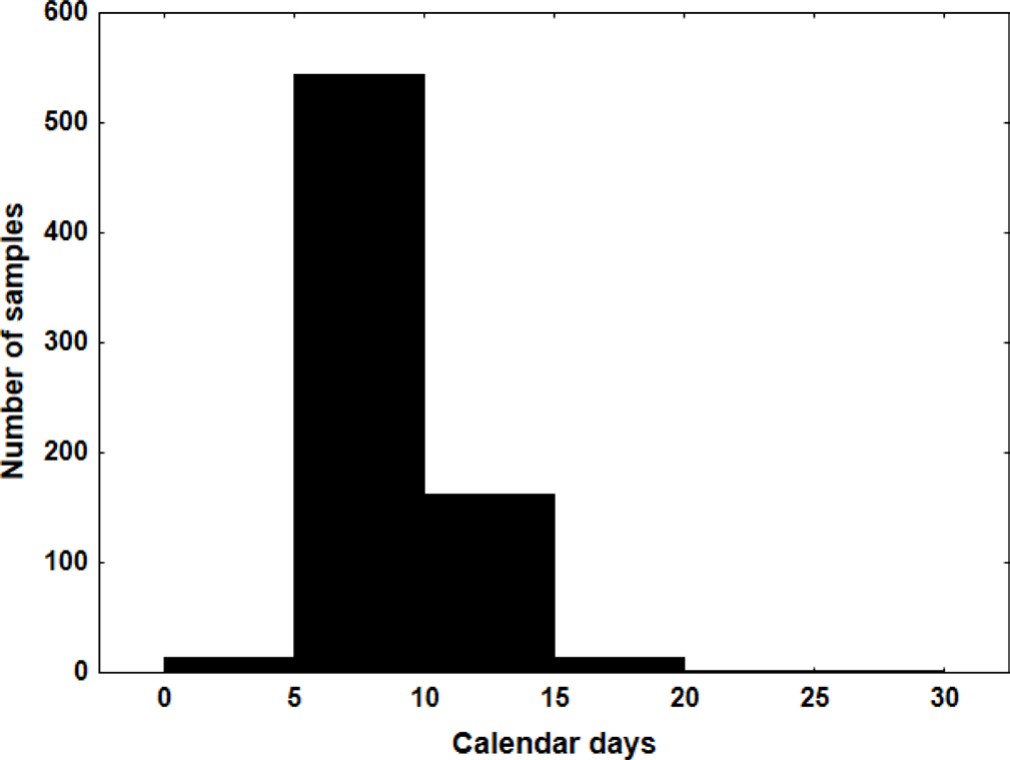
Histogram of the turnaround time measured in calendar days

It is important that clinically relevant targets are reported within a clinically useful timeframe. International guidelines recommend that RAS results should be available within 7–10 working days of receiving the specimen in the testing laboratory [6–9]. We tracked the turnaround time (TAT) by retrieving status data from the laboratory information system. The median TAT between reception of the sample in the laboratory and report release was 8 calendar days. For 76% of the cases, the report was released within 10 calendar days after receiving the samples (Figure 2). Only 17 cases (2%) had a TAT above 10 working days, which was due to samples requiring reanalysis, interpretation difficulties and organizational delays.

### Overview of identified variants

The number of mutations per tumor ranged from 0 to 5 (mean 1.6). In the majority of the cases (534/727; 73%) only 1 or 2 mutations were detected. In 77 cases (11%) no mutations were found in any of the analyzed regions.

**Figure 3:**
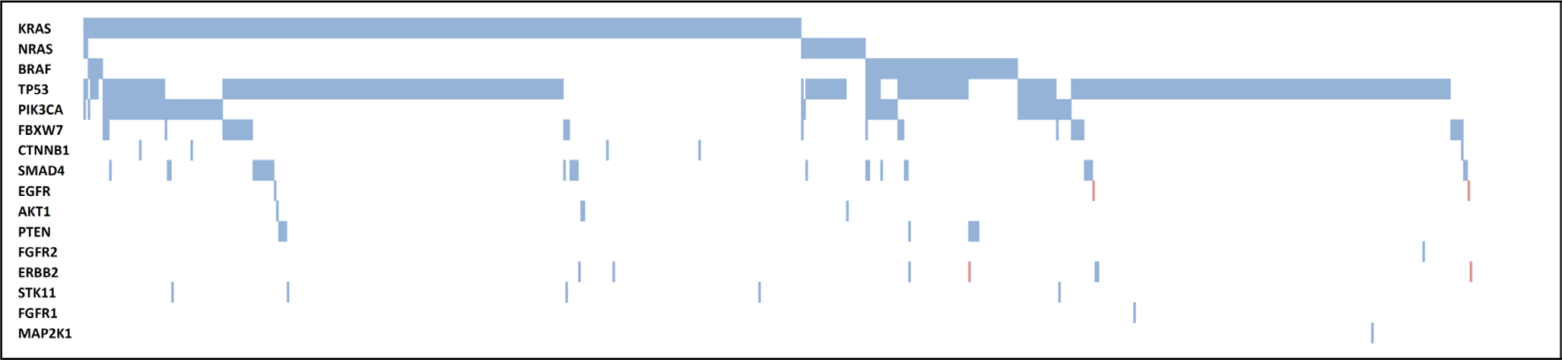
Molecular profile of CRC samples Molecular alterations in different genes (rows) are indicated for each CRC sample (columns). A full square indicates that a mutation was found (in the gene), a hatched square indicates that an amplification was found (in the gene), whereas an empty square indicated that no mutation was detected (in the gene).

The most frequent mutations were found in TP53 (62%) and KRAS (46%) (Table 2 and Figure 3). Of successfully sequenced cases, 563 potentially actionable mutations were identified in 464 patients (64%), including 343 KRAS mutations, 32 NRAS mutations, 78 BRAF mutations, 101 PIK3CA mutations, 4 AKT1 mutations, 3 ERBB2 mutations, 1 EGFR mutation, and 1 MAP2K1 mutation. The frequencies of these variants detected by NGS were consistent with frequencies reported in public databases (www.cbioportal.org; http://cancer.sanger.ac.uk/cosmic).

**Table 2:** Summary of the potentially actionable mutations identified (*N* = 563) and the percentage of patients with mutations in each gene

Gene	Number of mutations				% patients with mutations^**^
	PRIMARY	METASTASIS	UNKNOWN	TOTAL	
KRAS^*^	**268**	**50**	**25**	**343**	**46.1% (335/727)**
p.G12V/D/A/S/C/R/F	189	33	12	234	
p.G13D/R/S/C	41	6	7	54	
p.L19F	0	0	1	1	
p.Q22K	1	0	2	3	
p.A59T	1	0	1	2	
p.Q61K/H/L	7	2	0	9	
p.K117N	4	2	0	6	
p.A146T/V/G/P	25	7	2	34	
**NRAS**	**26**	**4**	**2**	**32**	**4.4% (32/727)**
p.G12V/D/S/C	8	1	1	10	
p.G13R/D/C/V	5	1	0	6	
p.A59T	0	1	0	1	
p.Q61K/L/H/R	13	1	1	15	
**BRAF**	**63**	**11**	**4**	**78**	**10.7% (78/727)**
p.R462I	0	0	1	1	
p.G466A/E/R	3	0	0	3	
p.G469E	1	0	0	1	
p.D594G/N	4	1	0	5	
p.F595L	1	0	0	1	
p.V600E	54	10	3	67	
PIK3CA^*^	**81**	**15**	**5**	**101**	**13.8% (100/727)**
p.E542K/V	9	2	1	12	
p.E545K/G/Q	38	1	1	40	
p.Q546P/K/L/H/R	11	3	1	15	
p.Q564P	1	0	0	1	
p.Y1021C	1	0	0	1	
p.H1047R/Y	21	9	2	32	
**AKT1**	**3**	**1**	**0**	**4**	**0.6% (4/727)**
p.E17K	3	1	0	4	
**ERBB2**	**3**	**0**	**0**	**3**	**0.4% (3/727)**
p.D762Y	1	0	0	1	
p.V777L	1	0	0	1	
p.V842I	1	0	0	1	
**MAP2K1**	**1**	**0**	**0**	**1**	**0.1% (1/727)**
p.K57N	1	0	0	1	
**EGFR**	**0**	**1**	**0**	**1**	**0.1% (1/727)**
p.D761N	0	1	0	1	

^*^Double mutations in KRAS: 2 cases with G12D and G13D; 1 case, each with G12V and G13S, G12A and G13D, G12A and G12V, G12A and A146T, G13D and A146T, Q22K and L19F. Double mutations in PIK3CA: 1 case with E545K and H1047Y.

^**^Note that some patients had mutations in more than one gene.

The detection of mutations by NGS showed high sensitivity. Eighty-three, 89 and 91% of the cases with <10%, 10–50%, and >50% tumor cell content, respectively, showed at least one mutation. Furthermore, around 50% of cases showed KRAS and/or NRAS mutations for each category of tumor cell content (27/54 in cases with <10% tumor cell content; 278/553 in those with 10–50% tumor cells; or 59/117 in cases with >50% tumor cells).

### Gene amplification detection

**Figure 4:**
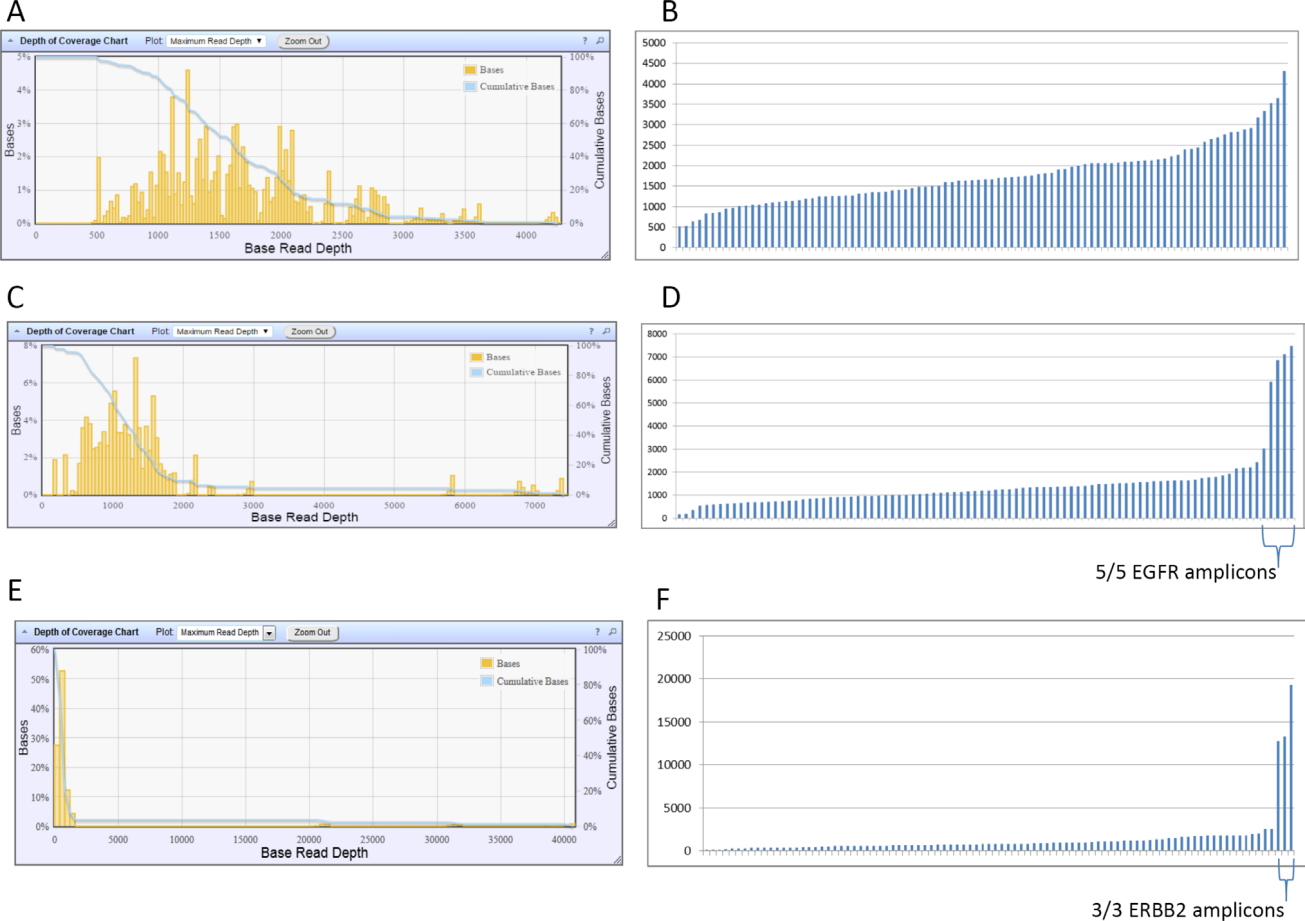
Histograms of the distribution of base read depth (**A**, **C**, **E**) and of the distribution of amplicon read depth (**B**, **D**, **F**) for a “typical” case (A, B) without gene amplification, for an EGFR-amplified case (C, D) and for an ERBB2-amplified case (E, F).

Using coverage analysis, potential EGFR or ERBB2 amplifications were suggested for 2 and 2 patients, respectively. Indeed, in 2 cases the mean coverage for the 90 amplicons was of 1,458× and 1,413×, whereas the mean coverage of the 5 EGFR amplicons was of 6,086× and 12,508×, respectively, suggesting the presence of an EGFR gene amplification (Figure 4). For the 2 other cases, the mean coverage for the 90 amplicons was of 1,366× and 1,329×, whereas the mean coverage of the 3 ERBB2 amplicons was of 6,432× and 15,019×, respectively (Figure 4). For these 4 cases, the suggested amplification was confirmed by ISH (Supplementary Figure 1).

## DISCUSSION

A major advantage of NGS over traditional mutation detection methods is its ability to screen multiple mutations in multiple genes simultaneously without the need to perform several sequential tests. Several studies have already validated the use of NGS and its superiority in terms of sensitivity and speed compared to traditional methods [10–13]. In our own experience, for tests including more than two to three different hotspots, NGS is faster and requires less DNA than would be needed for traditional methods.

In the present study, we report a large series of CRC patients for whom the tumor was prospectively analyzed in a daily practice setting using targeted NGS. Of 741 consecutive CRC patients, the molecular profiling of the tumor was successful for 98%.

Coverage analysis allowed us to suggest high-level amplification of ERBB2 or EGFR genes, as already described [14]. However, this technology has to be further validated for copy number variation analysis in order to determine its sensitivity and specificity. In the present study, all suggested amplifications were confirmed by ISH. However, we can’t be sure that all amplifications (especially low level amplification) are detected.

One of the critical steps in implementing new technology in routine testing is TAT. International guidelines recommend that RAS results should be available within 7–10 working days of receiving the specimen in the testing laboratory for >90% of the samples [7–9]. Our median TAT is of 8 calendar days and tends to decrease with years (median TAT of 11 calendar days in 2013 and 8 calendar days in 2015). The TAT is often increased by the fact that samples need to be pooled in order to fill a run once a week. If a request arrives in the laboratory one day too late for DNA extraction, the test is postponed to the next week. A potential solution would be to perform more than one run per week. However, this clinical scenario would be possible only if the number of samples to test increased in order to fill out the run and be cost-effective.

The molecular profiling of CRC tumors reported in the present study is similar to that reported in the literature and public databases [6, 15–20]. We are able to identify potentially actionable mutations for 464 patients, most of them carrying KRAS and/or NRAS mutations (365). According to international guidelines [2, 4], the presence of a RAS mutation is a contraindication to anti-EGFR therapy. Nevertheless, the discovery of particular mutations in rare genes can cause a change in the treatment of the patient by including him/her in a clinical trial or medical need program. According to our data, 14% of the patients (99/727) qualify for enrolment in a clinical trial. However, given the fact that the patients in the present study come from different institutions, we are not able to record the outcome of the patients or the exact proportion of patients enrolled in a clinical trial.

In conclusion, the requirements for implementation of a new test in daily practice include that (i) the test must be performed on routine samples with low DNA content, (ii) the test results must be delivered rapidly, and (iii) the test results must be accurate and facilitate clinical decision-making. The present study shows that targeted NGS is suitable for use in clinical daily practice. This new technology is very attractive because it provides a mutational profile of a tumor, leading to precision medicine.

## MATERIALS AND METHODS

### Samples

Tumor samples from 741 patients from 14 different institutions (private laboratories, academic and non-academic centers) for whom molecular testing was requested by medical oncologists were analyzed in a daily routine practice. Data were prospectively collected. DNA was extracted from FFPE tumor samples, after macrodissection of the tumor area, using the QIAamp FFPE tissue kit (Qiagen, Antwerp, Belgium) according to the manufacturer’s instructions. The H&E stained slide from the same block, previously reviewed by a pathologist who circled the tumor area and evaluated the tumor percentage, was used as a guide for the macrodissection. The DNA obtained was quantified using the Qubit^®^ fluorometer in combination with the Qubit^®^ dsDNA HS assay kit (Life Technologies, Gent, Belgium).

### Next-generation sequencing

NGS was performed as previously described [13, 21]. Briefly, 10 ng of DNA was amplified using the Colon and Lung Cancer panel (Ampliseq™, Life Technologies) in order to sequence 1825 hotspot mutations in 22 genes (90 amplicons) including AKT1, ALK, BRAF, CTNNB1, DDR2, EGFR, ERBB2, ERBB4, FBXW7, FGFR1, FGFR2, FGFR3, KRAS, MAP2K1, MET, NOTCH1, NRAS, PIK3CA, PTEN, SMAD4, STK11, TP53. Sequencing was performed on a PGM™ sequencer. The raw data were analyzed using the torrent suite software (v4.0 to v5.0 - Life Technologies). The coverage analysis was performed using the coverage analysis plug-in. Cases for which the number of mapped reads was <100,000 and/or the average base coverage was <500× were considered as non-informative. Mutations were detected using the Variant Caller plug-in (v4.0 to v5.0) with low stringency settings (Life Technologies). In the variant list obtained, we considered a variant as authentic if the variant coverage was at least 30× and if the variant frequency was at least 4% [13, 21]. Moreover, each mutation was verified in the Integrative genome viewer (IGV) from the Broad Institute (http://www.broadinstitute.org/igv/). Only mutations reported in the COSMIC (Sanger Institute Catalogue of Somatic Mutations in Cancer) database (http://www.sanger.ac.uk/cosmic) were taken into account, and silent or intronic mutations were not reported. A case was considered to be without mutation if the tumor content was at least 10% tumor cells, if the case was considered informative, and if no mutation was detected. Moreover, hotspot regions in KRAS (exons 2, 3, 4), NRAS (exons 2, 3, 4) and BRAF (exon 15) were manually verified for each case in the Integrative Genome Viewer (IGV) from the Broad Institute (http://www.broadinstitute.org/igv/). Mutation detection sensitivity was controlled for by including either the AcroMetrix^™^ Oncology Hotspot Control (Life Technologies) or the Tru-Q HDx^™^ Reference Standard (Horizon Discovery, Cambridge, UK) in each of the Ion PGM^™^ runs.

The method used (including DNA extraction, sequencing and data analysis) is ISO 15189 certified.

### Gene amplification evaluation

Coverage analysis of the NGS data allowed us to evaluate potential high level amplifications of ERBB2 (HER-2) and EGFR genes. Gene amplification was suggested when coverage analysis revealed a deviation in depth of coverage for all the amplicons covering ERBB2 or EGFR (Figure 4). For these cases, *in situ* hybridization was performed to confirm the gene amplification.

### *In situ* hybridization

When coverage analysis suggested an amplification of ERBB2 (HER-2) or EGFR genes, *in situ* hybridization (ISH) was performed for confirmation. Fresh cut, 4-µm sections of FFPE tissue were submitted to dual-color ISH using probe sets. HER-2 fluorescence ISH was performed using the PathVysion HER-2 DNA Probe Kit (Abbott, Wavre, Belgium) according to the manufacturer’s instructions. EGFR chromogenic ISH was performed using the ZytoDot^®^ 2C SPEC EGFR/CEN 7 Probe (Zytovision, Bremerhaven, Germany) according to the manufacturer’s instructions.

## SUPPLEMENTARY MATERIALS FIGURE



## Abbreviations

CRC: Colorectal Cancer
NGS: Next-generation sequencing
FFPE: formalin-fixed paraffin-embedded
TAT: turnaround time
