# Supplementary material for: Clinical application of targeted next-generation sequencing for colorectal cancer patients: a multicentric Belgian experience
Source: Oncotarget. 2018 Apr 17;9(29):20761–8. doi: 10.18632/oncotarget.25099 (PMC5945518; doi:10.18632/oncotarget.25099)
Supplement: Supplementary file 1 [file oncotarget-09-20761-s001.pdf]

Clinical application of targeted next-generation sequencing for colorectal cancer patients: a multicentric Belgian experience

SUPPLEMENTARY MATERIALS

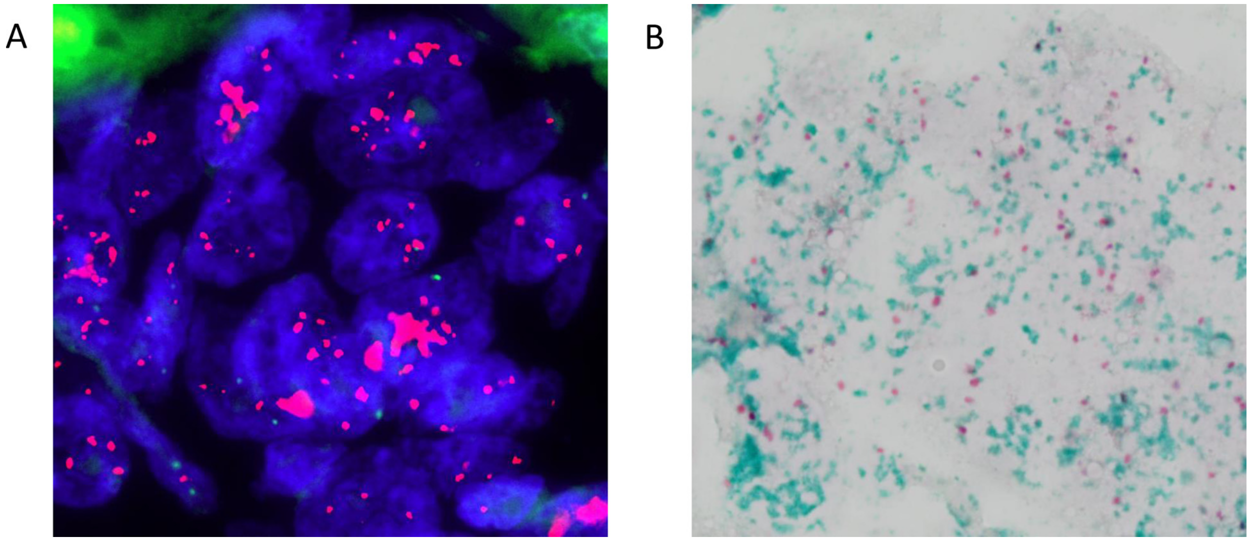

Supplementary Figure 1: cases suggestive of a gene amplification were confirmed by ISH. (**A**) ERBB2 amplified case (dual color FISH – green: CEP17/red : ERBB2 gene – original magnification ×1000). (**B**) EGFR amplified case (dual color CISH – red: CEP7/ green: EGFR gene – original magnification: ×1000).
